# Beyond Earth: Harnessing Marine Resources for Sustainable Space Colonization

**DOI:** 10.3390/md22110481

**Published:** 2024-10-24

**Authors:** Marco F. L. Lemos

**Affiliations:** MARE—Marine and Environmental Sciences Centre, ARNET—Aquatic Research Network Associated Laboratory, ESTM, Polytechnic of Leiria, 2520-641 Peniche, Portugal; marco.lemos@ipleiria.pt

**Keywords:** bioregenerative life support, biomanufacturing, circular economy, marine organisms, space biotechnology, sustainable space exploration

## Abstract

The quest for sustainable space exploration and colonization is a challenge in its infancy, which faces scarcity of resources and an inhospitable environment. In recent years, advancements in space biotechnology have emerged as potential solutions to the hurdles of prolonged space habitation. Taking cues from the oceans, this review focuses on the sundry types of marine organisms and marine-derived chemicals that have the potential of sustaining life beyond planet Earth. It addresses how marine life, including algae, invertebrates, and microorganisms, may be useful in bioregenerative life support systems, food production, pharmaceuticals, radiation shielding, energy sources, materials, and other applications in space habitats. With the considerable and still unexplored potential of Earth’s oceans that can be employed in developing space colonization, we allow ourselves to dream of the future where people can expand to other planets, not only surviving but prospering. Implementing the blend of marine and space sciences is a giant leap toward fulfilling man’s age-long desire of conquering and colonizing space, making it the final frontier.

***Beyond Earth, the Final Frontier***

*As humanity aims for space exploration, the dream of colonizing space pushes us to go beyond Earth’s limits. However, achieving this goal requires us to tackle the challenges of sustaining life in the vastness of space. As our ambitions grow, the pursuit of self-sufficiency in space calls for innovative solutions to ensure both survival and success. This necessity opens the door to exploring blue biotechnology as a key tool for sustainable space colonization. By tapping into the resources of Earth’s oceans and applying that knowledge to outer space, we are driven to explore new possibilities at the intersection of human creativity and the wisdom found in marine life, setting the stage for shaping the future of human existence beyond Earth’s boundaries.*

## 1. Exploring the Intersection of Space Biotechnology and Marine Resources

### 1.1. Pioneering Sustainable Biotechnological Solutions for Space Exploration

Within the immense realm of space exploration, the quest for sustainable solutions acts as a guiding principle as humanity ventures outside the limits of Earth. Within this framework, the integration of biotechnology and marine resources arises as a compelling frontier, providing novel avenues toward sustainable space settlement. The exceptional ability of marine organisms to adapt and recover, influenced by the long evolutionary history of Earth’s oceans, represents a valuable reservoir of solutions to overcome the challenges of space colonization [1,2,3,4].

Central to this undertaking is the advancement of bioregenerative life support systems, which act as the fundamental support for sustainable space habitats. Marine algae, known for their rapid growth rates and ability to facilitate photosynthesis, are prominent participants in this field [5,6]. Photosynthesis enables these algae to produce oxygen necessary for human respiration and also engage in carbon dioxide fixation, which is vital for preserving a balanced atmospheric composition in closed-loop life support systems [7]. Additionally, marine microorganisms demonstrate exceptional abilities in the treatment of wastewater and recycling of nutrients, providing effective solutions for resource management in limited space settings [8,9].

As mankind envisions embarking on extended space expeditions to remote celestial bodies, the need to employ resources in a sustainable manner becomes more evident. The incorporation of marine aquaculture into space habitats shows potential for meeting the dietary requirements of astronauts by offering a sustainable and nutrient-dense food supply in the challenging space environment [10,11,12]. Furthermore, the investigation of chemicals obtained from marine sources for pharmaceutical purposes presents new opportunities for tackling health issues in outer space, including the development of new drugs and the practice of tissue engineering [13,14,15].

The integration of biotechnology and marine resources into the endeavor for sustainable space exploration marks a notable transition, ushering in a new epoch of ingenuity and adaptability [1]. The feasibility of achieving sustainable space colonization is growing as researchers continue to push the boundaries of scientific knowledge and technological innovation, driven by a desire for exploration and the plentiful resources of the oceans.

### 1.2. Embracing Resource Utilization and Sustainability in Long-Duration Space Missions

With the expansion of human activities beyond the boundaries of Earth’s protective environment, the need for sustainable management and use of resources becomes challenging as it is of the utmost importance. The increasing impracticality of regular resupply missions from Earth necessitates a paradigm shift toward self-sufficiency and resilience in long-duration aerospace missions [16]. In contemporary space exploration, the principle of sustainability is present in all elements of mission planning and implementation, propelling the advancement of cutting-edge technologies and approaches for resource utilization [17,18].

**Figure 1:**
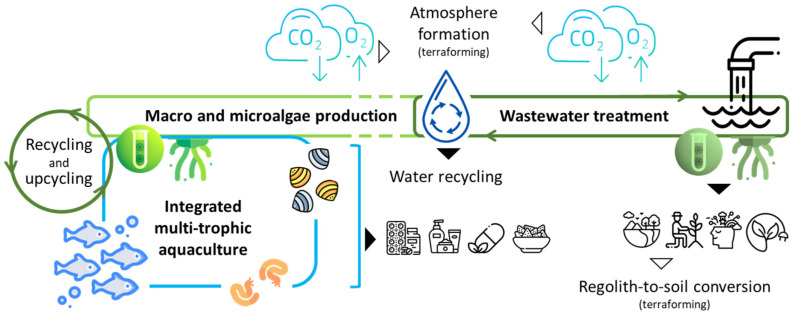
Schematic representation of a marine-based life support system designed for space habitats, illustrating the integration of microalgae for oxygen production, aquaculture for food supply, and wastewater treatment, all functioning within a closed-loop ecosystem to maximize resource efficiency and sustainability. This system promotes terraforming efforts by creating a self-sustaining ecosystem, thereby reducing reliance on external supplies.

The cornerstone of long-duration space missions’ sustainability lies in closed-loop systems, which involve meticulous management, recycling, and regeneration of resources to reduce waste and optimize efficiency [3,19,20] (Figure 1). For example, water reclamation technologies are vital in guaranteeing the accessibility of drinkable water for crew consumption and other necessary requirements [21,22]. Pioneering filtration systems and state-of-the-art purification methods empower astronauts to retrieve water from diverse sources, such as wastewater and even atmospheric moisture, thereby guaranteeing a continuous supply throughout the entire duration of the mission [6,23,24].

**Figure 2:**
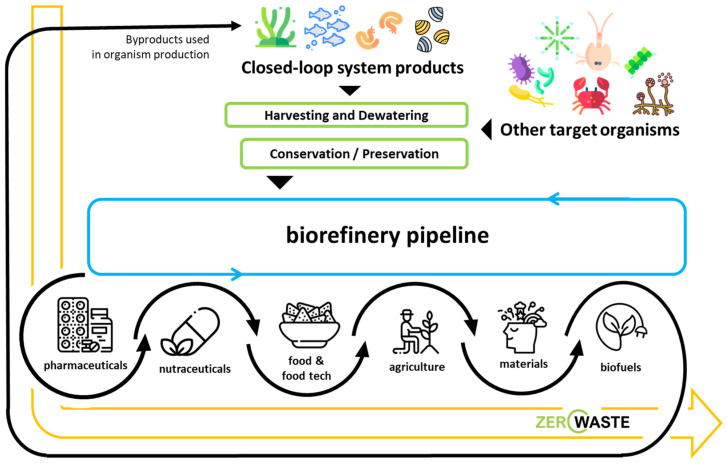
Schematic overview of a marine-based biorefinery system pipeline featuring sequential added-value products aimed at achieving a zero-waste goal, underscoring the principles of sustainability and resource efficiency, essential for enabling life in space.

Waste management is an essential aspect of sustainable space exploration, featuring technologies specifically developed to convert and recycle waste materials into useful resources, promoting circularity and a paradigm shift from waste to byproducts [3,25,26] (Figure 2). Recycling systems that can transform organic waste into compost dense with nutrients or even edible biomass enhance the closed-loop ecology of space habitats, decreasing dependence on external replenishment and mitigating environmental consequences [8,26]. Furthermore, endeavors to advance additive manufacturing methods, commonly known as 3D printing, by utilizing recycled materials facilitate the production of tools, spare parts, and even habitat components as needed, thereby further improving the sustainability of missions [27,28].

Energy production is a key area of focus in sustainable space exploration, with an increasing focus on renewable energy sources like solar and, potentially, nuclear power [29,30]. But marine resources may also offer promising opportunities, particularly through the exploration of biofuels derived from marine algae. Algae, for instance, have the potential to produce biofuels through photosynthesis, converting sunlight (or other light source) into usable energy, which could complement solar energy systems. Additionally, marine microorganisms are being studied for their ability to produce biohydrogen, a clean energy source, through biological processes [31]. Thus, marine-based biofuels could one day play a role in powering long-duration missions and supporting habitats.

Exposure to cosmic radiation in space presents a distinct challenge in terms of radiation protection, as it poses substantial hazards to the health of astronauts. Sustainable radiation shielding solutions utilize cutting-edge materials and design principles influenced by nature, such as biomimetic shielding derived from marine organisms or sophisticated composite materials designed for optimal effectiveness and low weight [32,33,34]. With the integration of radiation shielding into spacecraft design and habitat construction, mission planners seek to reduce the impact of cosmic radiation and guarantee the safety and welfare of crew members during their space travel beyond Earth [35].

Essentially, adopting resource-efficient and sustainable practices in extended space missions signifies a fundamental change in our approach to space exploration. Through the utilization of closed-loop systems, waste recycling, renewable energy, and others, alongside biorefinery processes designed for zero waste and complete resource utilization [36] (Figure 2), humanity establishes the foundation for a sustainable human existence beyond the confines of Earth, enabling future generations to engage in exploration and economic prosperity in outer space.

## 2. Marine Resources for Bioregenerative Life Support Systems

### 2.1. Algal Oxygenation: Harnessing Marine Algae for Life Support and In Situ Production

In the demanding space environment, where the generation of vital resources is vital for maintaining human life, marine algae become indispensable assets for producing oxygen in closed-loop life support systems. Utilizing the photosynthetic capabilities of marine algae, namely microalgae, offers a viable and environmentally compatible approach for generating oxygen in space environments [37]. Marine algae exhibit exceptional photosynthesis capabilities, harnessing sunlight to transform carbon dioxide and water into oxygen and organic compounds. This process serves the dual purpose of restoring the oxygen levels required for human respiration and aiding in the elimination of carbon dioxide, thus promoting a more stable atmospheric composition in enclosed space settings [38]. For example, *Chlorella vulgaris* may generate around 1.6 grams of oxygen per liter of culture daily under optimal conditions [39], while *Spirulina platensis* can remove 1.58 g of CO_2_ per liter of culture per day while producing oxygen and abating NOx [40].

The suitability of different species of marine algae in space habitats has been investigated, focusing on important aspects such as growth rate, biomass yield, and nutrient requirements [6,41,42]. Microalgae, including *Dunaliella salina*, *Chloromonas brevispina*, and *Chlorella vulgaris*, as well as the unicellular flagellate *Euglena*, show a vast potential as oxygen producers in space due to their fast growth rates and high photosynthetic efficiency [43,44,45,46]. Moreover, microalgae can be efficiently grown with reduced resources, such as water and light, which makes them highly suitable for closed-loop systems that prioritize resource conservation. *Chlorella vulgaris*, in particular, has been extensively researched for its high biomass productivity and resistance to environmental stressors [47]. When cultivated using Martian regolith and synthetic urine, *C. vulgaris* maintained a high protein content, reaching 37.1% of dry weight [48]. Under optimized conditions using LED-based photobioreactors, *C. vulgaris* achieved a biomass density of approximately 20 g/L and productivity of up to 2.11 g per liter of culture per day [49]. These findings suggest that microalgae, especially *C. vulgaris*, could be viable candidates for oxygen production and as a food source in space habitats, addressing critical challenges in long-term space missions.

In addition to their oxygen-producing capabilities, the cultivation of marine algae can be further enhanced by leveraging local planetary resources, such as regolith and atmospheric CO_2_, which are crucial for in situ production, a recognized approach to making future crewed missions sustainable [50]. This integration aligns with in situ resource utilization (ISRU) strategies, positioning algae as a crucial component of sustainable life support systems in extraterrestrial environments. Recent studies have demonstrated the potential for cultivating microalgae and cyanobacteria using Martian resources for life support systems on Mars. Experiments with *Spirulina*, *Chlorella vulgaris*, and *Haematococcus pluvialis* have shown successful growth using simulated Martian regolith and synthetic urine as nutrient sources [48,50,51]. These cultivation methods not only support biomass production but also can enhance nutritional profiles, with increased carbohydrates, proteins, and carotenoids observed in some cases [48]. Furthermore, a low-pressure atmosphere mimicking Mars’s composition (96% N_2_ and 4% CO_2_) at 100 hPa has been found suitable for cyanobacterial growth, supporting both autotrophic and diazotrophic processes [52]. The CO_2_-rich atmosphere and microgravity conditions may even improve productivity compared to Earth conditions [50]. By exploiting these local resources, the ability to produce marine organisms in situ can significantly reduce space missions’ dependence on supplies transported from Earth, thereby extending mission duration and enhancing the feasibility of long-term human settlement.

Continued efforts are being made to enhance the growth conditions for marine algae in space settings by investigating variables such as light intensity, nutrient availability, and cultivation vessel design in order to achieve the highest possible productivity and efficiency [6,47,53]. Furthermore, investigation is concentrated on the integration of algae cultivation systems with other elements of life support systems, such as wastewater treatment and nutrient recycling or other organism rearing, in order to establish mutually beneficial closed-loop ecosystems that reduce resource wastage and improve overall sustainability [54,55].

Through utilizing the intrinsic capacities of marine algae to generate oxygen, space agencies and researchers will extend space missions and establish sustainable human settlements beyond the confines of Earth [6]. Further developments in algal cultivation methods and their integration with life support systems offer the potential to safeguard the physical and mental health of astronauts as they explore deeper into the universe.

### 2.2. Utilization of Marine Microorganisms for Wastewater Treatment and Nutrient Recycling

In order to ensure the long-term viability of human settlement and maintain life support systems in space habitats, it is essential to effectively manage wastewater and recycle nutrients to both eliminate them and also use them as new byproducts [8,56]. The varied metabolic characteristics of marine microorganisms make them very promising for the management of wastewater and the recycling of nutrients through the efficient decomposition of organic waste and the recycling of essential nutrients [57,58].

The decomposition of organic matter and the recirculation of nutrients are crucial roles performed by aquatic microorganisms, including bacteria, archaea, and fungi [59]. In the context of space exploration, these microorganisms can be employed to perform similar functions in closed-loop life support systems, transforming waste objects into resources that can be reused by astronauts [5,8,60].

An important application of marine microorganisms in space settings is the purification of wastewater, whereby these microorganisms decompose organic pollutants and remove contaminants from wastewater pools [61]. Marine microorganisms, such as *C. vulgaris* and *Scenedesmus obliquus*, have demonstrated the ability to remove up to 95% of ammonia nitrogen and 80% of phosphorus from wastewater through nutrient uptake and assimilation [62,63]. Additionally, through aerobic and anaerobic digestion, denitrification, and nitrification processes, microorganisms like *Nitrosomonas* and *Nitrobacter* can convert harmful nitrogenous compounds such as ammonia into less toxic forms, reducing nitrogen levels by up to 98% [64].

Marine microorganisms may play a crucial role in the nutrient recycling process by converting organic waste into very valuable resources such as compost or biogas. In the process of breaking down organic matter, microorganisms release important nutrients like nitrogen, phosphorus, and potassium. These nutrients could be used to help plants and algae grow in space agriculture systems or added back into nutrient solutions for hydroponic farming [8,65].

Continuing research is being conducted to assess the performance of marine microorganisms in space environments by examining factors such as temperature, pH, and nutrient availability in order to enhance microbial activity and efficiency [6,45,66]. Changes in bioreactor technology make it possible to control the growth of microorganisms in space settings, offering a scalable and environmentally friendly method for treating wastewater and recycling nutrients [6,67,68].

International space agencies and academia are harnessing the metabolic capabilities of marine microorganisms to develop innovative techniques for treating wastewater and reusing nutrients. This allows for the creation of self-sustaining physical environments in space and the facilitation of lengthy space expeditions to remote locations [5,6,66].

### 2.3. Potential of Marine Aquaculture for Food Production in Space Habitats

Marine aquaculture offers a promising solution for sustainable food production in space habitats, offering a renewable and nutritionally rich food source for astronauts. By harnessing the vast potential of marine organisms, space agencies and researchers are exploring innovative approaches to meet the dietary needs of crew members over protracted space missions [10,11].

Being recognized as rich sources of essential nutrients, including proteins, vitamins, and minerals, marine species are therefore quite valuable assets for a well-balanced diet for astronauts [69]. Additionally, marine aquaculture systems can be especially designed to operate within closed-loop ecosystems, enabling the recycling and use of waste products produced by aquaculture activities to support other elements of life support systems, such as plant development or wastewater treatment [12,70]. Milanez et al., 2003 [71] report marine recirculating aquaculture systems achieving an annual production of 90 kg/m^3^ for gilthead seabream, consuming only 10% of the seawater used in flow-through systems. A global study found that RAS yields were particularly high, with marine systems yielding approximately 5 times more than freshwater systems on average [72]. Notably, an alternative closed bioconversion system for tilapia achieved production rates exceeding 500 tons/ha/year with minimal water exchange [73]. Additionally, microalgae-based systems can contribute 30–60% of dietary protein needs in a closed-loop system, further enhancing the sustainability of space missions [74].

Scientists are investigating different types of species for aquaculture in space, paying attention to variables such as speed of growth, nutritional content, and environmental requirements. Sea bass, tilapia, trout, and salmon are being evaluated for their capacity to flourish in controlled settings, offering a consistent supply of low-fat protein [11,75,76]. Researchers are also examining shellfish such as shrimp, mussels, and oysters for their rapid growth and rich nutritional value [10], while some bivalves and other marine invertebrates may also function to sequester pathogens [77].

Seaweeds, or marine macroalgae, represent another promising avenue for food production [78]. These highly nutritious organisms are appreciated for their abundance of protein, vitamins, and minerals, which makes them flexible components in space meals [79]. Furthermore, seaweeds are well-suited for growing in closed-loop systems because they only need seawater (nutrients) and sunlight, requiring minimal resources, which can be further coupled to integrated aquaculture systems including other higher-order organisms [80].

Current research endeavors are focused on enhancing the cultivation and harvesting of marine organisms in space settings. These efforts aim to investigate various aspects, including water quality, light intensity, and cultivation vessel design, in order to achieve maximum productivity and efficiency [10,11,54]. Recent developments in aquaculture technology, including recirculating aquaculture systems and integrated multitrophic aquaculture, allow for the sustainable and scalable cultivation of marine organisms in space environments [81,82].

Space agencies and researchers are utilizing marine aquaculture to develop robust and self-sustaining food production strategies for human settlement in extraterrestrial environments. By means of ongoing research and innovation, marine aquaculture systems hold the potential to provide astronauts with a wide range of nourishing food choices during prolonged space missions, enabling humanity to explore and flourish.

## 3. Marine Pharmaceuticals and Biomaterials

### 3.1. Exploration of Marine-Derived Compounds for Drug Discovery and Biomedical Applications

The exploration of marine-derived compounds for drug discovery and biomedical applications is an exciting and rapidly growing field in pharmaceutical research. Using the great diversity of marine habitats, researchers are finding novel medicinal molecules and innovative biomedical technology. A great variety of bioactive chemicals produced by marine organisms—including bacteria, fungus, algae, and invertebrates—show promise in treating a broad spectrum of diseases and medical disorders [83,84,85].

Many times displaying great pharmacological action against different disease targets, these marine-derived molecules with their special chemical structures and modes of action—often different from those of terrestrial organisms—make them especially important. Their originality raises their potential as brand-new therapeutic candidates [85]. Alkaloids, peptides, polyketides, terpenes, and steroids are a few examples of marine-derived compounds with therapeutic potential that marine organisms produce for a variety of ecological purposes, such as defense, communication, or reproduction [86,87]. These marine natural products have shown promise as anticancer agents, antimicrobial agents, anti-inflammatory agents, analgesics, and cardiovascular drugs, among others [84,88,89,90].

Beyond drug development, marine-derived chemicals are finding use in other spheres of medicine, including tissue engineering, regenerative medicine, and biomedical imaging [91,92]. For example, marine-derived biomaterials, such as collagen and chitosan derived from marine sources, have been investigated for their use in scaffolds for tissue regeneration and wound healing [93,94]. Furthermore, under development for advanced imaging systems like magnetic resonance imaging (MRI) are marine-derived contrast agents, including natural sepia melanin, a major component of the cuttlefish ink [95].

Collaboration between scientists from various disciplines like marine biology, organic chemistry, pharmacology, and biomedical engineering is vital for researching marine-derived compounds for drug discovery and biomedical uses. Advancements in high-throughput screening, metabolomics, and bioinformatics have allowed for the uncovering and examination of new marine-derived compounds with possible medicinal uses [96]. Despite the significant potential of marine-derived chemicals, their advancement for medical use faces multiple challenges. These tasks involve ensuring the continuous collection of marine organisms, separating and refining bioactive compounds, understanding how they work, and improving their pharmacological properties for specific extraterrestrial uses and particularities, providing new opportunities for the development of novel therapies and technologies to address unmet medical needs and enhance human health [85,96,97].

### 3.2. Use of Marine Biomaterials for Tissue Engineering, 3D Bioprinting, and Nanotechnologies in Space

Marine biopolymers, obtained from abundant marine resources including algae, crustaceans, and fish have attracted considerable interest for their potential to be used in tissue engineering and 3D bioprinting [98,99]. Known for their biocompatibility, biodegradability, and structural versatility, these biopolymers are highly suitable for the production of scaffolds and bioinks used in tissue regeneration and organ fabrication [100,101], providing suitable mechanical support and enabling a favorable microenvironment for cell growth and tissue regeneration [95]. These biomaterials include alginate, carrageenan, chitosan, hyaluronic acid, and collagen and gelatin derived from fish, having several benefits including biocompatibility, printability, biodegradability, and mechanical integrity [99,101]. More than that, biomaterials that come from marine sources often have biological properties that make them better for healing and regeneration, such as antimicrobial, anti-inflammatory, and pro-regenerative properties [102].

Increasingly, marine biopolymers are acknowledged as a valuable resource for the advancement of 3D bioprinting, namely in the field of tissue engineering in space. Their capacity to imitate the extracellular matrix renders them highly advantageous in fabricating intricate and adaptable tissue designs that can be customized to fulfill the particular requirements of astronauts in outer space [99,103]. The biopolymers conform to the principles of closed-loop resource utilization, which is essential for long-term space missions [104,105]. The application of renewable and sustainable marine biopolymers in 3D bioprinting shows potential for delivering tailored and readily available medical solutions to astronauts. This methodology can effectively tackle complexities associated with restricted storage capacity and supply networks in extraterrestrial settings. Furthermore, the progress made in biotechnology pertaining to space exhibits promise for the establishment of circular bioeconomies beyond the confines of Earth [4].

In order to fully exploit the potential of marine-derived biopolymers for tissue engineering and 3D bioprinting in space, researchers must overcome several obstacles. These challenges encompass finding the best marine-derived scaffolds in terms of their mechanical properties and degradation rates so that they can meet the needs of different types of tissue; making 3D bioprinting technologies more precise and scalable so that they can make more complex and accurate tissue constructs; and making sure that bio-printed structures will stay stable and work properly in the harsh conditions of microgravity [101,106]. To achieve successful translation of marine biopolymer-based solutions from the laboratory into real-world applications in regenerative medicine and space-based biotechnology, it is essential to overcome these technical obstacles [106]. In spite of these problems, ongoing research is pushing the development of marine biopolymer-based solutions, which is making it possible for revolutionary advances in regenerative medicine and biomedical research on Earth and in space.

Diatoms, a class of microalgae, also offer significant potential in the context of space exploration due to their ability to biomineralize, positioning these organisms as promising candidates for advancing sustainable materials in extraterrestrial environments [107]. The silica-based cell walls, known as frustules, possess a hierarchical nanostructure that exhibits light-trapping properties, making them useful for nanophotonic technologies [108,109]. Additionally, diatoms’ ability to synthesize inorganic solids has applications in creating semiconductors and heavy-duty bio-composites, which could be crucial in constructing durable materials for space habitats [110].

## 4. Marine Resources for Bioenergy and Radiation Protection

### 4.1. Fueling the Future: Marine Algae for Space Biofuel Production

Marine algae have become a highly promising source of biofuels in the quest for sustainable energy solutions for space exploration. They provide renewable and carbon-neutral alternatives to conventional fossil fuels [111,112]. Marine algae, commonly referred to as seaweeds or microalgae, exhibit several beneficial characteristics for the production of biofuels. These include fast rates of growth, a dense lipid content, and the capacity to flourish in a wide range of environmental conditions [113]

A highly promising characteristic of marine algae is their capacity to effectively transform solar energy into biomass by means of photosynthesis. Marine algae play a crucial role in carbon sequestration and greenhouse gas emission mitigation by absorbing carbon dioxide and releasing oxygen [114,115]. Furthermore, specific types of marine algae sequester substantial amounts of lipids, or oils, in their cells, which can be obtained and transformed into biodiesel by techniques like transesterification [116,117]. For example, some microalgae have been shown to produce 20–50% of their dry biomass in lipids, which can be converted into biofuels for energy production [118]. The abundant oil content of certain marine algae, along with their fast rates of growth, renders them a highly attractive source for the production of biofuels, particularly in the realm of space exploration, where limitations in energy and resources are of utmost importance [119,120,121].

The production of biofuels in space poses distinct difficulties in comparison to terrestrial applications, such as reduced resources, restricted environments, and the lack of atmospheric oxygen [122,123]. Nevertheless, marine algae possess inherent benefits that render them highly suitable for the production of biofuels in space environments. Marine algae can be grown at low resource requirements, such as seawater and nutrients, which makes them very suitable for the resource-limited environments of space [124]. Furthermore, marine algae can be cultivated in closed-loop systems, which allow for the recycling and utilization of carbon dioxide from life support systems for photosynthesis, optimizing their sustainability and efficiency [125]. The algae have the ability to efficiently absorb carbon dioxide (CO_2_), generate oxygen, purify water, and function as a dietary supplement in bioregenerative life support systems [36]. Several experimental systems, including AQUARACK, AQUACELLS, and OMEGAHAB, have been specifically developed and evaluated for the purpose of space investigations [44]. One notable scientific development is the Eu:CROPIS project, which integrates *Euglena* as an oxygen generator in conjunction with tomato plants [44]. These studies emphasize the capacity of algae to be used in sustainable closed-loop systems for extended space missions.

Current research endeavors are focused on enhancing the cultivation and processing of marine algae for the purpose of biofuel production in space settings. In particular, this means creating photobioreactors and closed-loop cultivation systems that work well in microgravity and allow precise control of environmental factors like light, temperature, and nutrients. According to Chowdury and coworkers [126], these advances have the potential to improve the productivity and composition of marine algae strains, maximizing their utilization as renewable fuel feedstocks. In addition, scientists are developing innovative and effective techniques for collecting and extracting organic matter from algae in space environments to transform it into biodiesel or other biofuel products [127,128].

### 4.2. Potential of Marine Organisms for Shielding against Cosmic Radiation

Among the most formidable obstacles to long-duration space exploration is cosmic radiation, which presents astronauts with significant hazards in the harsh space environment [129,130]. To reduce these hazards, scientists are undertaking investigations into novel methods of radiation shielding, and marine organisms have emerged as promising contenders for offering defense against cosmic radiation.

The potential applications of marine algae extend beyond biofuel production to include radiation protection for space exploration [131,132]. Marine species have developed diverse strategies to mitigate environmental stressors such as ultraviolet radiation, ionizing radiation, and oxidative stress [133,134,135]. Specific marine organisms, including certain types of algae, bacteria, and fungus, generate specialized secondary metabolites that have demonstrated radioprotective characteristics in terrestrial settings. These compounds may therefore offer radiation protection in space exploration, presenting sustainable solutions to the difficulties of long-term space missions [131]. As antioxidants and free radical scavengers, peptides, polysaccharides, and polyphenols help protect cellular structures and genetic material from the damage made by radiation [132,136]. Possible applications of photobioreactors in space environments include the cultivation of algae or other marine organisms to generate radioprotective chemicals. These compounds can then be included in astronaut food, beverages, or even integrated into the structural materials of the spacecraft or habitat.

An exemplary instance of marine organisms exhibiting apparent radioprotective characteristics is the red alga *Porphyra yezoensis*, more widely referred to as nori. There are several sulfated polysaccharides in nori, including porphyran, which has been shown in land-based studies to have antioxidant and anti-inflammatory properties. The integration of nori extracts into spacecraft shielding materials or dietary supplements is being explored by researchers as a means to augment the radiation protection provided to astronauts during space missions [137,138].

Research has indicated that certain microorganisms, namely halophiles such as *Halorubrum chaoviator* and *Synechococcus*, demonstrate elevated rates of survival when subjected to space conditions [139]. Averesch and coworkers [140] reported that the fungus *Cladosporium sphaerospermum* displayed augmented growth rates and potential radiation-shielding capabilities outside the International Space Station. *Deinococcus radiodurans* is a marine bacterium extremophile known for its remarkable ability to withstand high levels of ionizing radiation and other environmental pressures, making it a very promising option for radioprotection [141,142,143]. Researchers have looked into how this thermophile bacterium’s DNA repair system and antioxidant defenses could be used to protect astronauts from radiation. Furthermore, marine microorganisms, such as certain bacteria and archaea, produce pigments known as carotenoids, which are cornerstones in protecting cells from UV radiation and oxidative stress. Astaxanthin, fucoxanthin, and β-carotene are carotenoids that have shown antioxidant and radioprotective properties in terrestrial studies. They are now being evaluated for their potential application in shielding against radiation in space [144].

Marine organisms can provide radiation shielding not only through their direct radioprotective effects but also through indirect mechanisms, including the formation of biofilms [145,146]. Biological films are complex assemblies of microorganisms that attach to a matrix of extracellular polymeric substances (EPS). These biofilms development, articulated through quorum sensing, a bacterial communication mechanism, can alter the surface properties of the host, affecting the availability of light, gas, and nutrients [146,147]. Marine organisms, their biofilms, and the environment can synergistically influence the ecological interactions of the host, which may provide additional protective advantages against radiation exposure [146]. The objective of this field of research is to improve radiation shielding and reduce astronauts’ exposure to cosmic radiation by utilizing the inherent characteristics of marine organisms to create biofilms on spacecraft surfaces or novel prospect habitat walls and structures.

The potential for the use of marine organisms in radiation shielding processes in space exploration is considerable, although certain obstacles persist. These challenges include the identification and characterization of prospective radioprotective chemicals, the optimization of extraction and formulation methods, and the integration of marine-derived materials into spacecraft and habitats. Notwithstanding these hurdles, efforts are being made for the use of marine organisms as a sustainable and efficient method to protect astronauts and structures from cosmic radiation during extended space missions [137,148].

## 5. Challenges and Future Directions

### 5.1. Technical and Biological Challenges in Utilizing Marine Resources in Space

Utilizing marine resources for space exploration involves addressing a range of technical and biological challenges. These challenges span various aspects of marine resource utilization, such as cultivation, processing, integration into life support systems, and adaptation to the unique space environment.

Beyond the mere use of these resources for short travel, a key technical challenge in using marine resources in space is developing efficient cultivation and processing methods that work within the constraints of space habitats. Space environments have limited space, resources, and environmental control, requiring the design of compact, resource-efficient cultivation systems to produce enough marine biomass for applications like food, drugs, bioenergy, and radiation shielding.

Furthermore, the integration of marine resources into closed-loop life support systems requires careful consideration of nutrient cycling, waste management, and environmental sustainability [149]. Closed-loop systems aim to minimize resource waste and maximize efficiency by recycling and reusing waste products, but achieving this goal in space environments presents additional challenges due to the limited availability of resources and the need to maintain stable environmental conditions for biological processes [26].

An important biological obstacle in harnessing marine resources in space is comprehending and enhancing the development and metabolic processes of marine organisms under microgravity constraints. Biochemical processes such as cell growth, differentiation, and metabolism are influenced by microgravity, potentially affecting the productivity and functionality of marine biomass grown in space habitats [150]. To guarantee the successful cultivation and utilization of marine organisms in space, it is crucial to thoroughly assess and reduce the impact of cosmic radiation and other space environmental elements on their health and viability [150].

The long-term viability and adaptability of marine resource exploitation systems in space habitats rely on the capacity to sustain stable ecological dynamics and avoid imbalances or disturbances that may endanger the health and well-being of astronauts. Ongoing monitoring, management, and adjustment of cultivation systems are necessary to guarantee maximum performance and productivity throughout prolonged mission periods [151].

To overcome these technical and biological obstacles in using marine resources for space exploration, international cooperation among scientists, engineers, and space agencies will be essential. Furthermore, the endeavor will need novel methods for growing, cultivating, processing, and incorporating these resources into space environments. Researchers aim to overcome these obstacles in order to fully exploit the capabilities of marine organisms and their products to facilitate sustainable human settlement and progress in space exploration beyond our planet.

### 5.2. Strategies for Sustainable Resource Management and Ecosystem Preservation

As humanity extends its presence into outer space, it becomes imperative to effectively and responsibly oversee resources and safeguard ecosystems. The effective utilization of resources and preservation of the fragile equilibrium of space-based ecosystems require strategic approaches that guarantee the long-term sustainability of space habitats and reduce environmental consequences.

In space, a crucial approach for sustainable resource management is the use of closed-loop life support systems. These systems are designed to reduce waste and optimize resource use by recycling and regeneration processes. Within a controlled environment, these systems replicate natural ecosystems by cycling water, nutrients, and air, decreasing dependence on external replenishment missions and minimizing the ecological impact of space habitats [23].

Closed-loop life support systems, which incorporate various components like water reclamation systems, air revitalization systems, and food production systems, create self-sustaining ecosystems capable of sustaining human life indefinitely. Hydroponics, aquaponics, and algae cultivation are vital technologies in these systems, as they provide the necessary food, oxygen, and water recycling capabilities for extended space missions [5].

Integrating closed-loop systems with the preservation of equilibrium in space-based ecosystems is of utmost importance. Close monitoring and management of the interactions among ecosystem components, from lower- to higher-order levels of organisms, are necessary to sustain a stable and healthy planet. Furthermore, it is essential to implement preventive measures in order to reduce potential disturbances such as the introduction of invasive species, the spread of diseases, or toxic substance accumulation [152,153]. By using sustainable resource management and ecosystem preservation techniques, space agencies and researchers can guarantee the long-term feasibility of human settlement in space, advancing environmentally conscious and ecologically sustainable growth beyond Earth [1,154].

The development of advanced recycling and waste management technologies, including marine biorefineries, should be seen as a vital component of sustainable resource management in space. Technological advancements enable the conversion of waste materials into useful resources such as biofuels, biochemicals, and a myriad of bioproducts [25]. Innovations in technology, like 3D printing and additive manufacturing, can streamline the fabrication of tools, spare parts, and habitat components using recycled materials. This reduces the need for external resupply and minimizes waste generation [155]. These approaches have the potential to significantly enhance the development of closed-loop systems and promote the progress of a circular economy in space settings.

The long-term success of space settings and the progress of space exploration depend on the necessary implementation of sustainable resource management and the preservation of ecosystems. Through the integration of closed-loop life support systems, the maintenance of ecological balance, the practice of regenerative agriculture, and the advancement of recycling technologies, it is feasible for humanity to establish resilient and self-sustaining communities in orbital space.

### 5.3. Future Prospects and Potential Collaborations between Marine Science and Space Agencies

The integration of marine research and space exploration presents significant opportunities for the discovery of novel information, stimulation of technical advancement, and enhancement of the sustainability of human endeavors beyond the confines of our planet. Space agencies and marine research groups are continuing to look into how these two areas are connected. As a result, many possible partnerships and mutual benefits are becoming clear. This is leading to revolutionary progress in space biotechnology and other related fields. Collaboration between marine science and space organizations primarily focusses on the investigation and utilization of marine resources to facilitate human settlement and exploration in outer space. Marine species have exceptional adaptability and durability, which make them valuable resources and solutions for tackling issues in extended space missions, including food production, bioenergy generation, radiation protection, and biomaterial processing. Through using the knowledge and infrastructure of marine research institutions, space agencies can accelerate the progress and implementation of environmentally friendly technologies and methods for space exploration. In addition, the examination of marine habitats and ecosystems on Earth offers significant knowledge regarding the possible suitability for sustaining life on other celestial bodies, such as Mars and the frigid moons of the outer solar system. Earth’s analogous habitats, including deep-sea hydrothermal vents, ice-covered lakes, and extremely salty conditions, are used as model systems to investigate the possibility of life beyond our planet and guide the development of future space missions aimed at detecting extraterrestrial life [156,157]. The identification of novel bioactive chemicals and biomaterials in marine biotechnology presents fresh opportunities for the fields of space biotechnology and biomedical science. The chemical structures and pharmacological activity of marine natural products are intricate, making them suitable for use in drug development, cell scaffolds, stem cell therapy, and radiation protection in space missions. Collaboration among marine biologists, biochemists, and space agencies can together discover, develop, and implement novel marine-derived technologies for space applications. The future prospects of collaboration between marine science and space agencies are quite promising. Through the integration of their knowledge and resources, these departments have the potential to stimulate innovation, promote multidisciplinary research, and tackle urgent issues that confront both people and the earth. By harnessing this synergy, novel prospects for exploration, discovery, and sustainability on Earth and beyond can be further unlocked.

## 6. Conclusion Remarks: Forging a Path beyond Earth

The present work explored the domains of space exploration and marine research, emphasizing the crucial significance of marine resources in propelling humanity’s endeavors to extend beyond the confines of our planet. The collaboration across these fields offers extraordinary opportunities for pioneering, durability, and exploration, including the profound depths of our oceans and the immense expanse of outer space. The marine resources possess significant potential to effectively tackle the objectives of extended space missions and the establishment of sustainable space settlement. The uses of marine resources in space biotechnology are many and promising, ranging from using marine algae for oxygen production to investigating marine-derived chemicals for drug discovery and making use of marine biomaterials for tissue engineering [1].

Significant potential exists in the partnership between marine science and space agencies. By amalgamating knowledge from both disciplines via interdisciplinary study, technological advancement, and international collaboration, we can expand the limits of human discovery and establish a viable future beyond our planet. Successfully achieving a self-sustaining human presence in space depends on the integration of marine resources with space technology. This methodology not only sustains the existence of life in outer space but also enhances our comprehension of the position of humankind within the expansive cosmos. At the core of this concept are closed-loop systems, which draw inspiration from the robustness of marine ecosystems. These systems efficiently coordinate the management, recycling, and regeneration of resources to establish sustainable habitats capable of supporting human life indefinitely, hence reducing waste and optimizing utilization. The concept of establishing a self-sustaining human presence outside of Earth, accomplished by the collaboration of marine science and space technology, illustrates a daring and motivating frontier in the realm of exploration and revelation. Through the use of marine resources and advancements in space technology, we initiate a dynamic process of creativity, adaptability, and environmental responsibility that holds the capacity to mold the future of mankind for future generations. The present era necessitates the incorporation of blue biotechnology with the investigation of the vast uncharted territory of space. At this point, blue biotechnology arises as a potent intellectual viewpoint, clarifying how humans might make a sustainable contribution to life outside the terrestrial boundaries. Progressions in marine science and space technology are approaching the point when the ocean bodies encircling the Earth will be the basis for humanity’s exploration into outer space. Through the utilization of microalgae for bioregenerative life support systems and the extraction of bioactive marine chemicals for pharmaceutical development, blue biotechnology is positioned to be a thrilling and revolutionary expedition toward uncharted territories of exploration, where limitations are no longer present and space emerges as the next realm to conquer and innovate within.

## Data Availability

Not applicable.
